# Pigmented paravenous retinochoroidal atrophy with acute angle-closure glaucoma and posterior subcapsular cataract: a case report

**DOI:** 10.1186/s12886-022-02355-5

**Published:** 2022-04-22

**Authors:** Yilin Sun, Jia Li, Li Yu, Yajuan Zheng

**Affiliations:** Department of Ophthalmology, The Second Hospital of Jilin University, Changchun, China

**Keywords:** Pigmented paravenous retinochoroidal atrophy, Retinitis pigmentosa, Acute angle-closure glaucoma, Posterior subcapsular cataract

## Abstract

**Background:**

Pigmented paravenous retinochoroidal atrophy (PPRCA) is a rare fundus disease characterized by the presence of osteoblast-like pigment, atrophy of retinal pigment epithelium (RPE), and choroid deposition along the large retinal veins.

**Case presentation:**

A 55-year-old Chinese female presented with right eye distention and bilateral vision loss. Osteocyte-like pigmentation and retinal choroidal atrophy distributed along the large retinal veins were seen in the fundus of bilateral eyes. The atrophy in the left eye was more severe compared to the right eye. The patient also presented with bilateral acute angle-closure glaucoma (AACG) and posterior subcapsular cataract (PSC) accompanied with anterior segmental manifestations, similar to the complications of retinitis pigmentosa (RP). The patient underwent ultrasound biomicroscopy (UBM), Humphrey field analyser (HFA), optical coherence tomography (OCT), fundus autofluorescence (FAF), fluorescein fundus angiography (FFA), electroretinogram (ERG), and electrooculography (EOG), all of which confirmed the aforementioned diagnose.

**Conclusion:**

PPRCA is a rare disease of unknown etiology. The patient in this case presented with complications similar to those of RP, and the two conditions may share a genetic basis. Further studies are needed to confirm this relationship.

**Supplementary Information:**

The online version contains supplementary material available at 10.1186/s12886-022-02355-5.

## Background

PPRCA is characterized by osteoblast-like pigmentation along the large retinal veins accompanied with atrophy of the outer retina and choroid. This condition is prevalent in men, and it is symmetrical in bilateral eyes [1]. The cause of PPRCA remains unknown, and there is no effective treatment. PPRCA can be diagnosed by visual field examination, fundus photography, optical coherence tomography (OCT), fundus autofluorescence (FAF), fluorescein fundus angiography (FFA), electroretinogram (ERG), and electrooculography (EOG). Since both RP and PPRCA have mutations in the CRB1 locus [2], some researchers have suggested that PPRCA is another form of RP [1]. Here, the patient was a 55-year-old Chinese woman who had asymmetrical fundus manifestations. She presented with AACG and PSC accompanied with PPRCA, which was consistent with the complications of RP. This case report might suggest a genetic link between PPRCA and RP.

## Case presentation

**Fig. 1:**
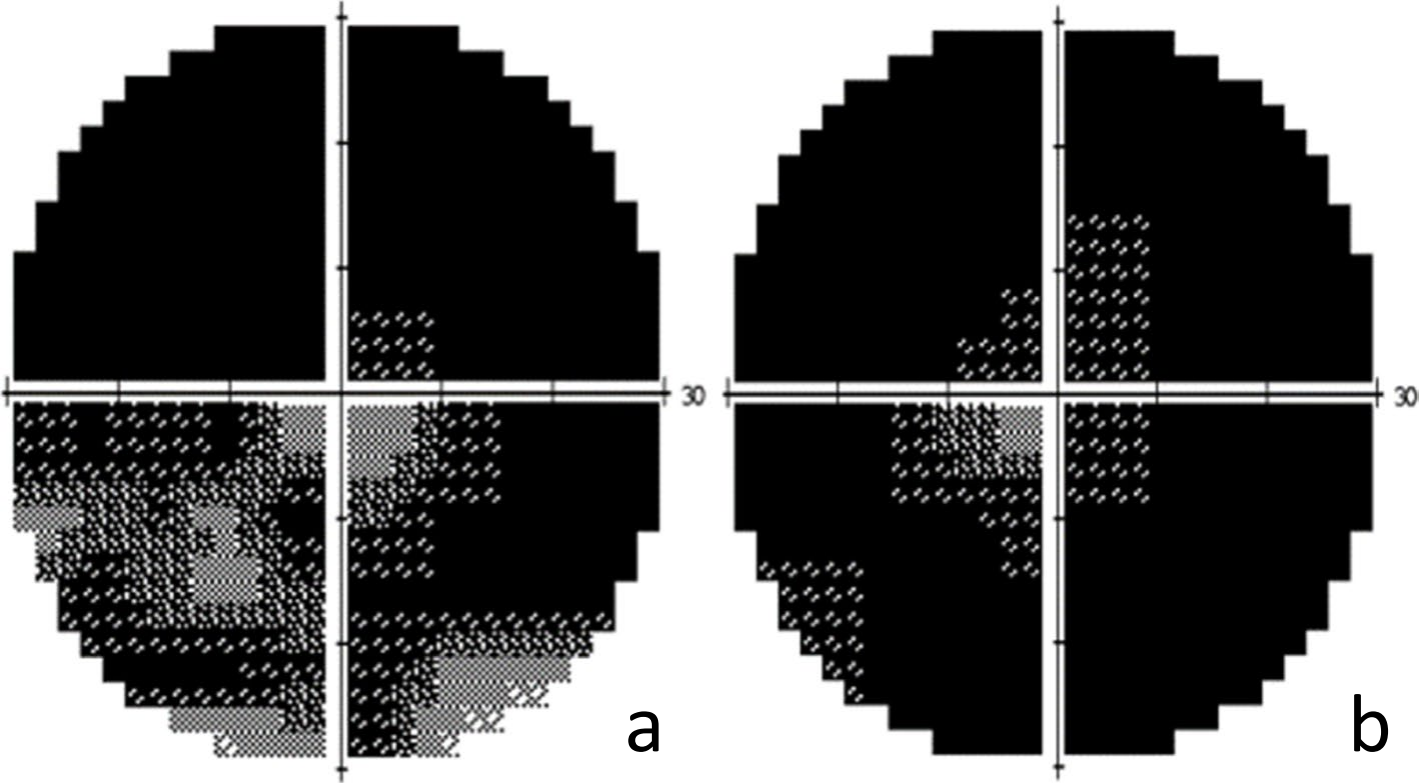
Humphrey visual field (HFA) showed a total vision loss in the OD (**a**) and the OS (**b**)

**Fig. 2:**
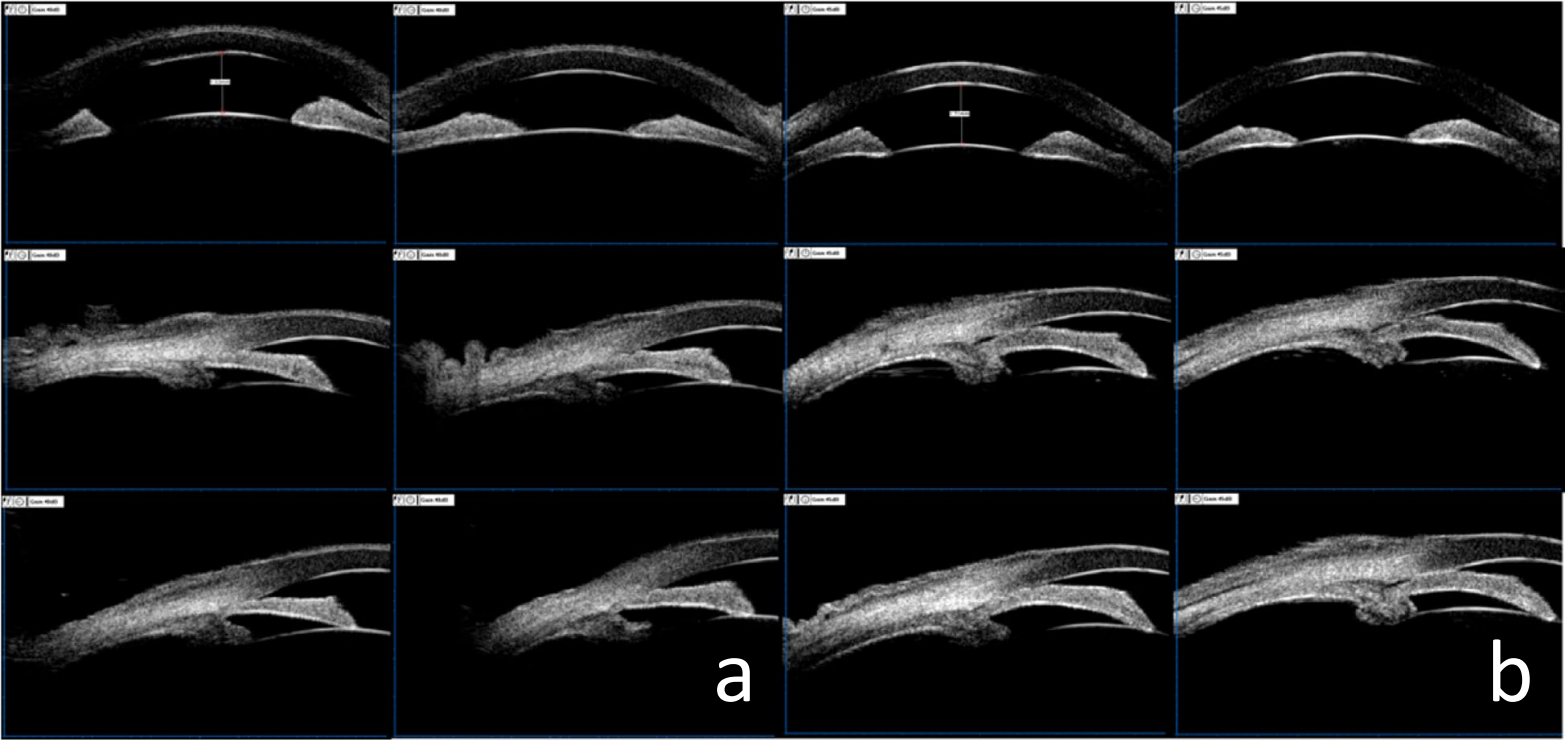
Ultrasound biomicroscopy (UBM) of the bilateral eyes. **a** Showing UBM in the OD with full circumferential closure of the atrial angle. **b** Showing UBM in the OS with narrowed atrial angle

**Fig. 3:**
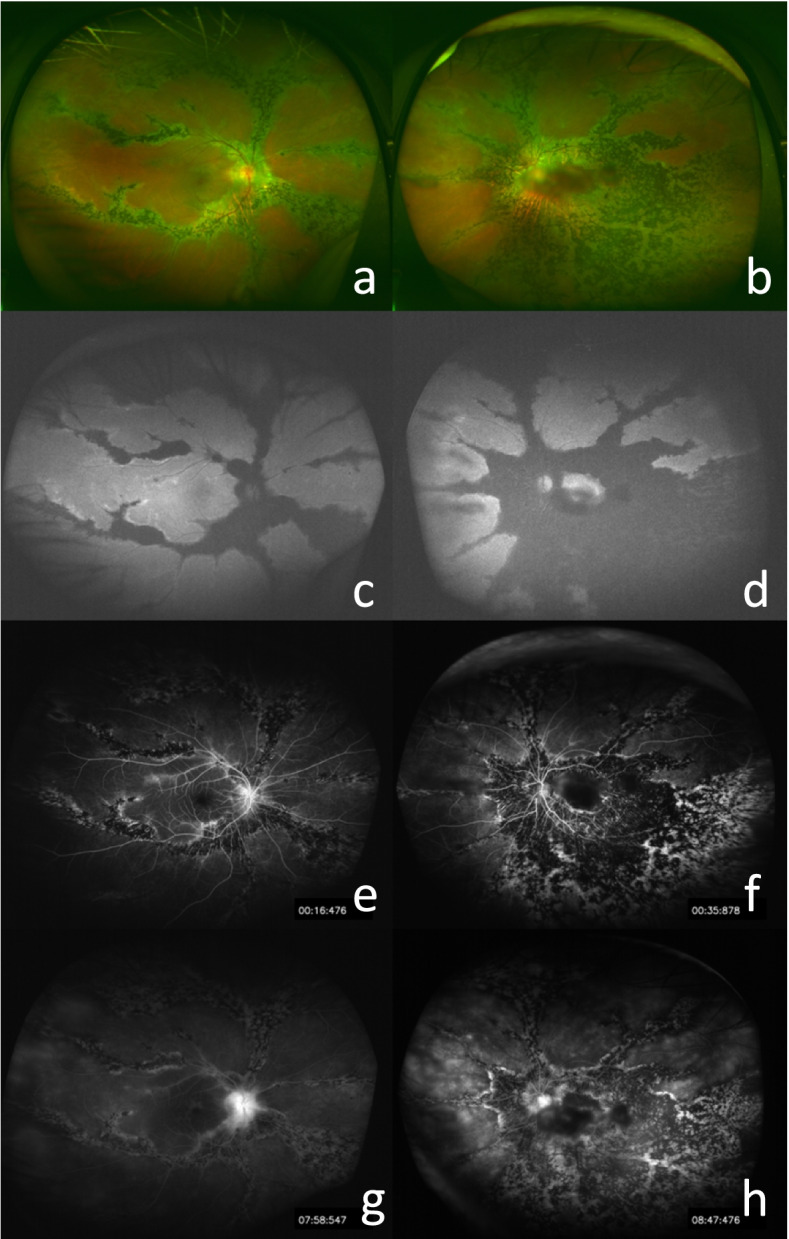
**a, b** Wide angle under-eye photography of the OD and OS showing retinal choroidal atrophy distributed along the large retinal veins, with large choroidal vessels visible around the optic disc. **c, d** Fundus autofluorescence of the OD and OS indicating significant low fluorescence surrounding the high fluorescence band corresponding to the atrophy zone. **e, f** Early fluorescein fundus angiography of the OD and OS showing transmitted fluorescence at the edges of atrophy, and fluorescence masking the pigmented areas. **g, h** Late FFA of the OD and OS showing weak fluorescence in the atrophic areas of both eyes, with some areas of fluorescence staining. Cataract occlusion in the macular region of the OS

**Fig. 4:**
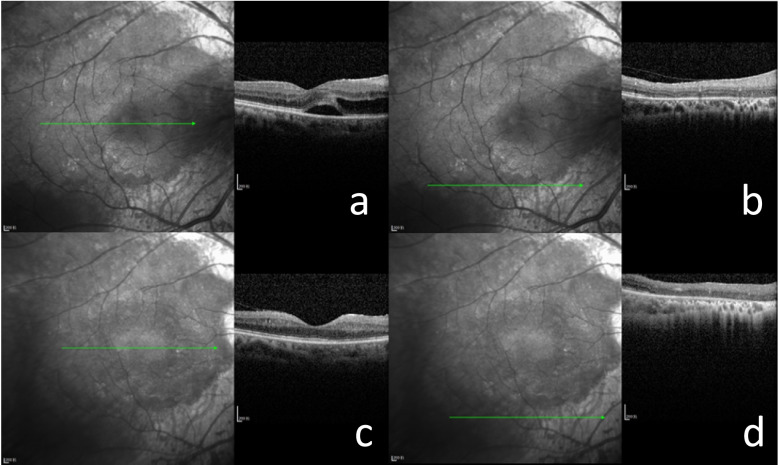
Optical coherence tomography (OCT) of the OD at diagnosis (**a, b**) and three months postoperatively (**c, d**). The hyporeflective signal was seen near the macula (**a**) and disappeared three months postoperatively (c). Atrophy of the retinal pigment epithelium (RPE) is shown. (**b, d**)

**Fig. 5:**
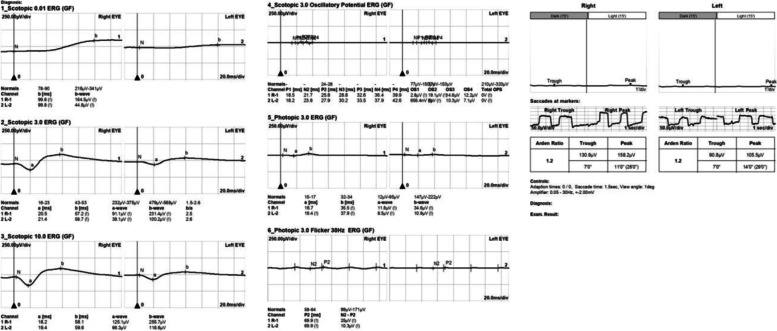
Electroretinogram (ERG) and Electro-oculography (EOG) of our patient. The photopic and scotopic amplitudes of a- and b- waves reduced in bilateral eyes, and the OS was more severe. The Arden ratio was 1.2 in bilateral eyes

A 55-year-old Chinese woman was first seen at our hospital due to distention and vision loss in her right eye (oculus dexter (OD)) for one month. She was diagnosed with PPRCA twenty years ago and had no history of night blindness, ocular or systemic diseases or any other inflammatory and infection diseases. Best corrected visual acuity (BCVA) at the first visit was 0.2 in bilateral eyes, and the anterior segmental examination of the bilateral eyes revealed characteristic symptoms of AACG and PSC such as shallow anterior chamber and posterior subcapsular cataract. Intraocular pressure (IOP) in the OD was 37 mmHg and 18 mmHg in the left eye (oculus sinister (OS)). In bilateral eyes, HFA demonstrated that the visual field was extremely poor. (Fig. 1 a to b) UBM results showed chamber angle closure in the OD and narrowing in the OS. (Fig. 2 a to b) Wide-angle fundus photography confirmed retinal choroidal atrophy and osteocyte-like pigmentation along the large veins in bilateral eyes, and large choroidal vessels could be seen in the area of atrophy near the optic disc. Large foci of atrophy was seen in the inferior temporal area of the OS. Further, FAF showed significant hypoautofluorescence in bilateral eyes corresponding to the area of atrophy, with some borders surrounding the jagged hyperautofluorescence, suggesting progressive RPE atrophy. In addition, there was a weak background fluorescence in the early atrophic area and transmitted fluorescence at the edge of atrophy that was affected by pigmentation. The pigmentation resulted in obscured fluorescence seen next to the veins. A small patch of obscured fluorescence was found in the posterior pole affected by posterior subcapsular cataract. In the late stage, fluorescence staining was seen in the atrophic areas. (Fig. 3 a to h) Analysis of OCT revealed a significant thinning of the retinal nerve fiber layer in the macula of the OD and detachment of the neuroepithelial layer in the macula. The outer retina in the atrophic area had thinned. There was no RPE in the area with severe atrophy and thinning of the choroid was confirmed. (Fig. 4 a to d) Results of the ERG showed a drop in photopic and scotopic amplitudes of a-and b-wave, more significantly in the OS. EOG revealed a bimanual Arden ratio of 1.2, which was below the standard value. (Fig. 5) The axial length of the OD was 21.09 mm and the OS was 20.15 mm. These findings lead to the diagnosis of PPRCA, AACG, and PSC in both eyes.

This patient underwent trabeculectomy combined with cataract phacoemulsification aspiration followed by IOL implantation in the OD and Nd-YAG laser iridotomy in the OS. Intraoperative dilatation of the pupil in the OD revealed that the patient’s lens suspensory ligament was relaxed. Three months after surgery, the patient’s BCVA was 0.6 in the OD and 0.2 in the OS, IOP was 17 mmHg in the OD and 13 mmHg in the OS. A further OCT showed the area of intraretinal capsule-like hyporeflective signal in the macula had disappeared, but the other fundus symptoms did not improve significantly. (Fig. 4 a to d) The patient was examined for the entire exome gene, and no ocularly significant mutation loci were identified.

## Discussion

PPRCA is a rare degenerative disease of the fundus of the eye with unknown etiology. It was originally discovered by Brown et al. during fundus examination of a male patient with mottled baldness [3]. Typically, PPRCA is characterized by RPE and choroidal atrophy along the large retinal veins with osteoblast-like pigmentation. The disorder is prevalent in men, and is always symmetrical in both eyes, develops slowly, rarely affects the macula, and it does not affect central vision. As a consequence, it is difficult to identify this condition in patients. There have been previous reports of PPRCA with strabismus [4], amblyopia [5], nystagmus [5], macular cystoid edema [6], macular hole [7], etc., but its relationship with concomitant diseases has not been reported. Huang et al. suggested that PPRCA is an additional, incomplete, self-limiting form of retinitis pigmentosa (RP) [1]. Mckay et al. identified heterozygosity for a val162-to-met (V162M; 604,210.0010) mutation within the fourth EGF-like domain of the CRB1 gene in PPRCA patients that was very close to the mutated *CRB1* gene locus in RP patients [2]. We have found no ocularly significant mutation loci. The genetic test we made could only detect known mutated loci, and there might be some unknown mutant loci that have not been identified in RP and PPRCA. Therefore, even if the test was negative, it might not prove that PPRCA is not associated with RP. The 55-year-old woman presented with severe PSC in bilateral eyes, posterior subcapsular cataract more in left eye than in the right eye, most probably due to a more serious PPRCA in the OS. During surgery, we discovered laxity of the lens zonules and suspensory ligaments, which seemed like RP complicated by cataract. We hypothesized that the PPRCA was linked to RP, and that the mechanisms of co-occurrence of AACG and PSC are similar to that of RP complicated by the latter two diseases.

This patient showed severe PSC in bilateral eyes and laxity of the lens zonules and suspensory ligaments, which is similar to the symptoms of RP with PSC. We speculate that PPRCA triggered RPE and choroidal atrophy, and the degenerated retinal tissue produced cytokines which broke through the damaged blood-retinal barrier and to injure the lens. The ensued long-term inflammatory state triggered laxity of the suspensory ligament [8–10]. This patient had a relaxed lens suspensory ligament, short axial length, and anterior segment crowding which predisposed her to AACG. The patient had a plasma exudate in the macular region of the OD, which was absorbed after IOP stabilization. PPRCA triggered RPE and choroidal atrophy, making the patient less tolerant to high IOP than an average patient with AACG. The fluid exuded from the choroidal vessels broke the blood-retinal intraocular barrier to accumulate in the neuroepithelium, which was gradually absorbed after IOP stabilization.

## Conclusions

In conclusion, we reported a rare case of PPRCA accompanied with AACG and PSC. We speculated that PPRCA might be an incomplete manifestation of RP. Given the poor understanding of PPRCA etiology, further genetic testing and more case studies are needed to investigate the relationship between PPRCA and RP.

## Supplementary Information

**Additional file 1.** Patient perspective.

## Data Availability

All date generated and analyzed during this study are included in this article.

## Abbreviations

PPRCA: Pigmented paravenous retinochoroidal atrophy
AACG: Acute angle-closure glaucoma
PSC: Posterior subcapsular cataract
OD: Oculus dexter
OS: Oculus sinister
RPE: Retinal pigment epithelium.
RP: Retinitis pigmentosa
BCVA: Best corrected visual acuity
UBM: Ultrasound biomicroscopy
HFA: Humphrey field analyser
OCT: Optical coherence tomography
FAF: Fundus autofluorescence
FFA: Fluorescein fundus angiography
ERG: Electroretinogram
EOG: Electrooculography
IOP: Intraocular pressure
