# Supplementary material for: Pigmented paravenous retinochoroidal atrophy with acute angle-closure glaucoma and posterior subcapsular cataract: a case report
Source: BMC Ophthalmol. 2022 Apr 22;22:184. doi: 10.1186/s12886-022-02355-5 (PMC9026714; doi:10.1186/s12886-022-02355-5)
Supplement: Supplementary file 1 — Additional file 1. Patient perspective. [file 12886_2022_2355_MOESM1_ESM.docx]

**Patient perspective**

I am a businessman and I need good vision to check my goods and communicate with my customers, but the gradual loss of vision over the years has reduced my quality of life, and the recent development of swelling and pain in my right eye has made me feel very painful. After the surgeon at the Second Hospital of Jilin University operated on me, my painful symptoms disappeared. After the surgery, I paid attention to eye hygiene and had regular follow-up examinations. My vision has improved and my life has become easier, which makes me feel very happy.
